# Lifestyle Interventions During Radiotherapy: A Scoping Review of Their Effects on Toxicity and Patient-Centered Outcomes

**DOI:** 10.3390/medsci14040501

**Published:** 2026-08-20

**Authors:** Anastasia Stergioula, Mavra Antiochou-Plexida, Marietina Giannoutsou, Eleni Gotsopoulou, Stamatina Karavopoulou, Ioanna Kitsou, Chrysovalantou Manolopoulou, Maria Pantelidi, Chrysanthi Pontiki, Panagiotis Plotas

**Affiliations:** 1 Postgraduate Program in Health Education, University of Patras, 265 04 Patras, Greecepplotas@upatras.gr (P.P.); 2 School of Medicine, National and Kapodistrian University of Athens, 115 27 Athens, Greece; 3 Department of Speech and Language Therapy, School of Health Rehabilitation Sciences, University of Patras, 265 04 Patras, Greece

**Keywords:** lifestyle intervention, radiotherapy, quality of life, fatigue, toxicity, exercise, nutrition, psychological

## Abstract

**Background/Objectives**: Lifestyle interventions may alleviate treatment-related toxicity and improve patient-centered outcomes during radiotherapy (RT). This scoping review mapped randomized evidence on lifestyle interventions delivered during RT, summarized reported outcomes, and identified evidence gaps. **Methods**: PubMed and Web of Science were searched for randomized controlled trials (RCTs) published from April 2016 to April 2026. Eligible studies included adults who received exercise, nutritional, psychological, combined, or multimodal lifestyle interventions during their RT treatment. Study characteristics, intervention details, and outcomes were charted and narratively synthesized. **Results**: Twenty-three RCTs were included. Exercise interventions were evaluated in 12 studies, nutrition in five, psychological in one, combined exercise-and-nutrition in two, combined nutrition-and-psychological in two, and multimodal interventions comprising all three components in one study. Breast, head and neck, and prostate cancers were most frequently represented. QoL, fatigue, and functional capacity were the most commonly assessed outcomes. Exercise interventions were most consistently associated with improvements in fatigue and functional capacity, whereas effects on QoL were less consistent. Nutritional interventions showed favorable findings for nutritional status and treatment-related toxicity. Psychological, combined, and multimodal interventions showed favorable findings for selected QoL, fatigue, nutritional, and psychological outcomes. Key evidence gaps included heterogeneous intervention protocols and outcomes assessment, limited evidence for non-exercise interventions and underrepresentation of several cancer populations. **Conclusions**: Lifestyle interventions delivered during RT may improve selected patient-centered and treatment-related toxicity outcomes. Exercise comprised the largest body of evidence, whereas other intervention types were less frequently evaluated. Heterogeneity across studies limits conclusions regarding optimal approaches. Further RT-specific studies using standardized intervention programs and comparable outcome measures are required to inform supportive-care delivery.

## 1. Introduction

Cancer remains a major global health burden and a leading cause of morbidity and mortality worldwide. Its incidence is projected to increase substantially, with the International Agency for Research on Cancer (IARC) estimating more than 35 million new cases annually by 2050, representing a 77% increase compared with 2022 [1]. Radiotherapy (RT) constitutes an essential component of cancer care, with approximately 50–60% of patients undergoing treatment during the course of their disease, either with curative or palliative intent [2]. Despite its well-established therapeutic efficacy, RT is frequently associated with treatment related toxicity that may adversely affect patients’ functional status, quality of life (QoL), and overall well-being.

In recent years, there has been growing interest in the role of non-pharmacological strategies, particularly lifestyle interventions, in mitigating treatment related toxicity and improving patient outcomes [3,4,5,6,7]. Lifestyle interventions incorporate a range of approaches, including structured exercise programs, nutritional modifications, and psychological support strategies such as stress management and mindfulness. These interventions are increasingly recognized as integral components of supportive cancer care [3,4]. However, the existing literature on lifestyle interventions in oncology remains highly heterogeneous. Many studies focus on specific cancer types or individual interventions, most commonly exercise, whereas other important domains, such as nutritional and psychological interventions, remain comparatively underexplored. Furthermore, much of the available evidence does not distinguish between different phases of cancer treatment, and there is limited synthesis specifically addressing the RT setting [5].

Given the complexity and diversity of the available studies, a comprehensive overview of the literature is warranted. The objective of this scoping review was to map the existing randomized evidence on lifestyle interventions in patients undergoing RT, categorize intervention types, summarize reported clinical outcomes, and identify key evidence gaps to inform future research and clinical practice. The review focused on toxicity and patient-centered outcomes rather than oncologic endpoints such as survival.

## 2. Materials and Methods

### 2.1. Review Protocol

A scoping review was conducted to map and characterize the available evidence on lifestyle interventions in adult patients with cancer undergoing RT treatment. This approach was considered appropriate given the heterogeneity of intervention modalities, cancer populations, and outcomes reported in the literature [8,9]. The review was conducted in accordance with the Joanna Briggs Institute (JBI) methodology [8] and reported following the Preferred Reporting Items for Systematic Reviews and Meta-Analyses Extension for Scoping Reviews (PRISMA-ScR) guidelines [10] (see Supplementary Materials, Table S1). The protocol was not prospectively registered, as this is not mandatory for scoping reviews. The review methodology was established a priori to enhance transparency and reproducibility.

The research question was developed using the Population–Concept–Context (PCC) framework [9]:

“What evidence exists regarding lifestyle interventions in adult patients with cancer undergoing radiotherapy?”.

The population involved adult patients with cancer receiving RT treatment, the concept of interest was lifestyle interventions, and the context was restricted to the RT treatment period.

### 2.2. Eligibility Criteria

Eligible studies involved adult patients (≥18 years) with cancer undergoing RT, either alone or in combination with chemotherapy (CHT), irrespective of whether CHT was delivered concurrently or sequentially, and evaluated lifestyle interventions delivered during the treatment period. Lifestyle interventions were defined as non-pharmacological interventions targeting health behaviors or supportive care and included exercise, nutritional, and psychological interventions. Studies were required to be published in English from April 2016 to April 2026. Although scoping reviews typically include diverse study designs, this review focused exclusively on randomized controlled trials (RCTs) to provide a methodologically consistent overview of the available evidence on lifestyle interventions during RT. Pilot and feasibility studies were excluded since their primary objective is to evaluate intervention feasibility rather than clinical effectiveness. Studies conducted exclusively pre- and/or post-RT were also excluded. For studies reporting outcomes at multiple time points, only data collected during the RT treatment period were extracted.

### 2.3. Search Strategy and Information Sources

A systematic literature search was conducted in PubMed and Web of Science electronic databases to identify eligible studies. The search strategy combined keywords and Boolean operators related to RT, cancer, and lifestyle interventions, as well as relevant outcomes including QoL, fatigue, and treatment-related toxicity. The following search string was adapted to the indexing requirements of each database: (“radiotherapy” OR “radiation therapy”) AND (“cancer” OR “oncology” OR “neoplasm”) AND (“exercise” OR “physical activity” OR “nutrition” OR “diet” OR “psychosocial” OR “psychological support” OR “lifestyle intervention”) AND (“Quality of Life” OR “QoL” OR “fatigue” OR “treatment related toxicity”). All terms were searched within study titles and abstracts.

### 2.4. Selection of Sources of Evidence

All records identified through database searches were collated and duplicates were removed prior to screening. Titles and abstracts were independently screened by two reviewers according to the eligibility criteria. The full texts of potentially eligible articles were subsequently retrieved and independently assessed by the same two reviewers. Any disagreements were resolved through discussion and consensus, with consultation from a third senior reviewer (A.S.) when necessary. The final list of included studies was reviewed and approved by all authors.

### 2.5. Data Charting Process

A spreadsheet was developed and used to systematically chart data from all included studies. Charted information included key study characteristics, intervention details, and outcomes related to treatment associated side effects. The charted data were summarized in a standardized table and synthesized narratively to identify patterns across the available evidence.

### 2.6. Data Items

The data charting process captured information on study characteristics, including author, year of publication, cancer type, and sample size. Lifestyle intervention-related data included intervention type and duration. Interventions were categorized according to their primary focus as exercise, nutrition, psychological, combined interventions comprising two lifestyle intervention components (e.g., exercise with nutritional or nutritional and psychological interventions), and multimodal interventions comprising all three components: exercise, nutritional, and psychological interventions. Outcome data were charted for treatment-related toxicity and patient-centered outcomes, including QoL, fatigue, nutritional outcomes, functional capacity, and psychological-related outcomes.

### 2.7. Synthesis of Results

A narrative synthesis was conducted to summarize the characteristics and findings of the included studies. Studies were grouped according to the primary type of lifestyle intervention, cancer type, reported outcomes, and key findings were summarized for each intervention category. To visually map the available evidence, evidence maps were constructed to illustrate the distribution of studies across intervention categories and cancer types, as well as the reported effects of lifestyle interventions across the assessed outcome domains. No quantitative synthesis or meta-analysis was undertaken, consistent with the objectives of a scoping review.

## 3. Results

### 3.1. Sources of Evidence

**Figure 1:**
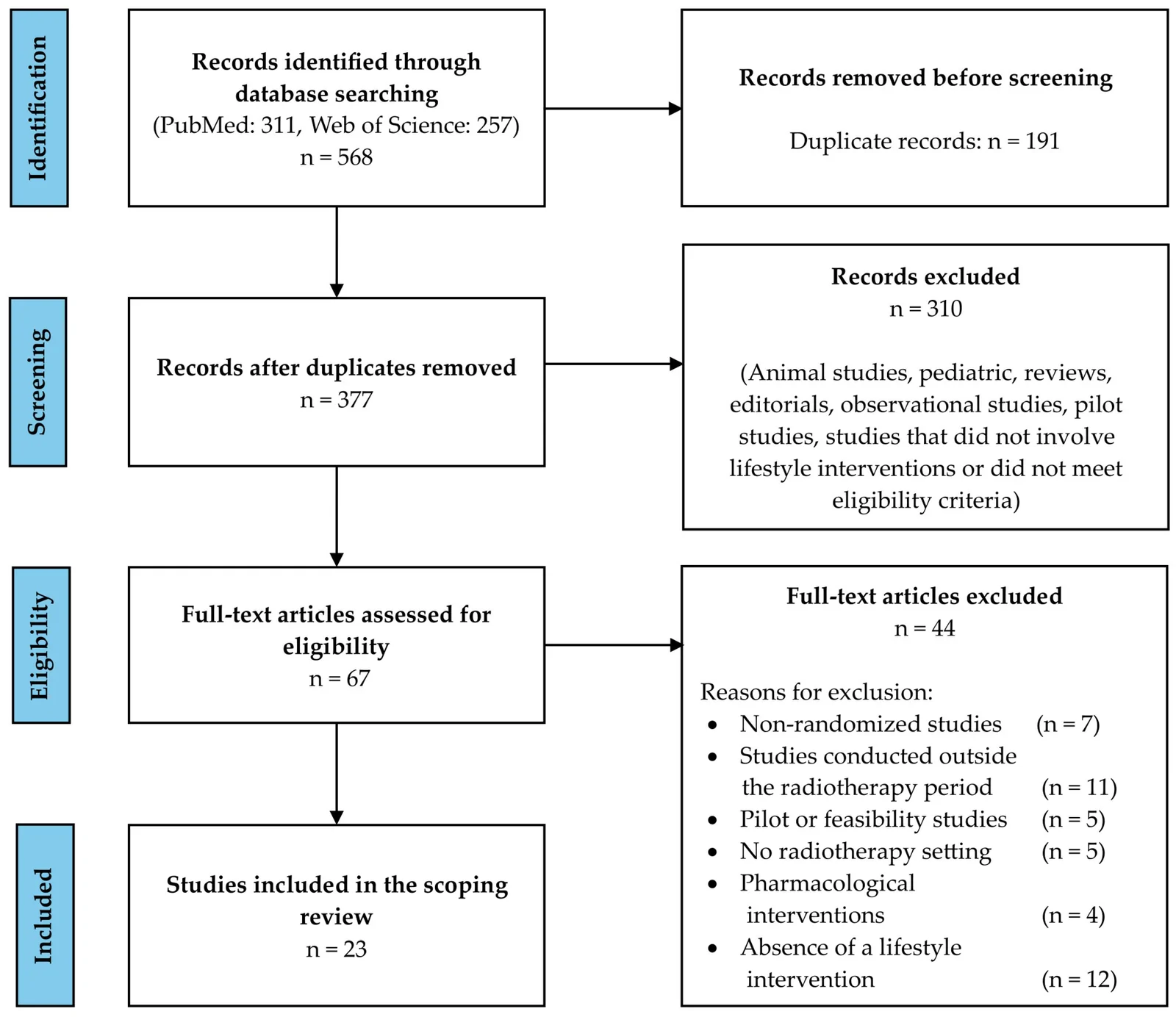
The PRISMA flow diagram of study selection.

The PRISMA flow diagram showing selection of the sources of evidence is presented in Figure 1. A total of 568 records were identified through the database searches. After the removal of 191 duplicates, 377 records were retained for title and abstract screening. During this stage, 310 records were excluded because they involved animal studies, review articles, editorials, observational studies, and pilot studies or they did not meet the eligibility criteria. Sixty-seven full-text articles were assessed for eligibility. Of these, 44 studies were excluded for the following reasons: non-randomized study design (*n* = 7), pilot or feasibility study design (*n* = 5), evaluation of pharmacological interventions (*n* = 4), absence of a RT setting (*n* = 5), interventions delivered outside the RT treatment period (*n* = 11), and absence of a lifestyle intervention (*n* = 12). Finally, 23 RCT studies were deemed eligible and were included in the scoping review.

### 3.2. Characteristics of Sources of Evidence

The characteristics of the included studies are summarized in Table 1. The studies were heterogeneous with respect to cancer type, sample size, intervention characteristics, intervention duration, and treatment modality (RT alone or in combination with chemotherapy (CRT)). Sample sizes ranged from 54 to 307 participants, while intervention duration varied from 3 to 50 weeks. Eleven of the 23 studies (48%) evaluated patients treated with RT alone, whereas the remaining 12 studies (52%) included patients receiving CRT.

**Figure 2:**
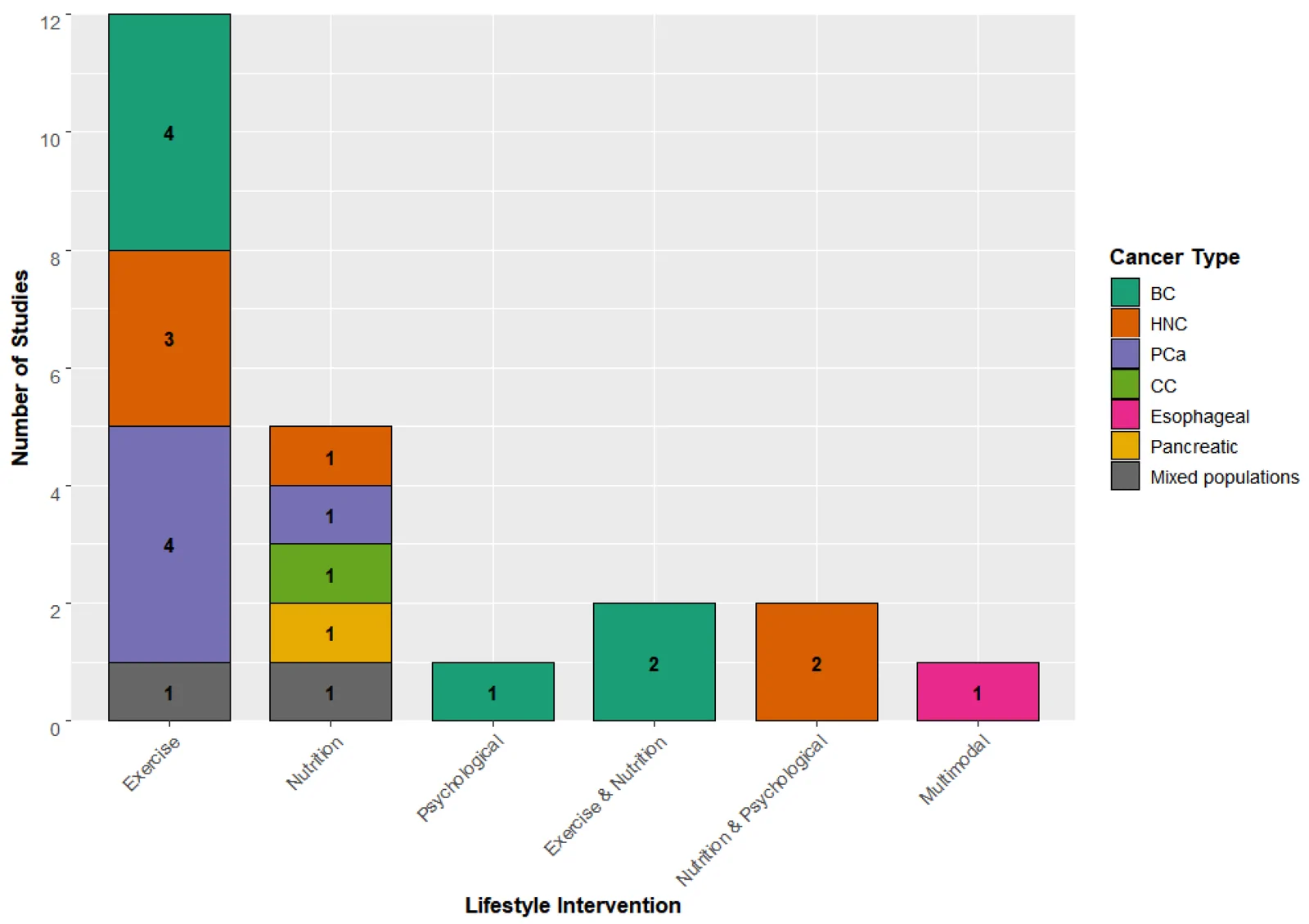
Number of included studies by lifestyle intervention, stratified according to cancer type.

The distribution of the available evidence according to the lifestyle intervention and cancer type is shown in Figure 2. Among the 23 included studies, 12 investigated the effect of exercise interventions, while five focused on nutrition interventions. One examined the effect of psychological intervention. Two evaluated combined exercise and nutrition interventions, two assessed combined nutrition and psychological interventions and one study investigated the effect of multimodal intervention. Exercise interventions accounted for nearly half (52%) of the included studies, whereas nutritional, psychological, combined and multimodal interventions were less frequently investigated.

Breast cancer (BC) was the most frequently studied cancer type (*n* = 7), followed by head and neck cancer (HNC) (*n* = 6) and prostate cancer (PCa) (*n* = 5) (Figure 2). The remaining studies included cervical cancer (CC) (*n* = 1), esophageal cancer (*n* = 1), pancreatic cancer (*n* = 1), and mixed cancer populations (*n* = 2). One mixed-population study included patients with HNC, BC, CC, and PCa, whereas the other enrolled patients with gynecological (GYN) and lower gastrointestinal (lower-GI) cancer undergoing pelvic CRT.

**Figure 3:**
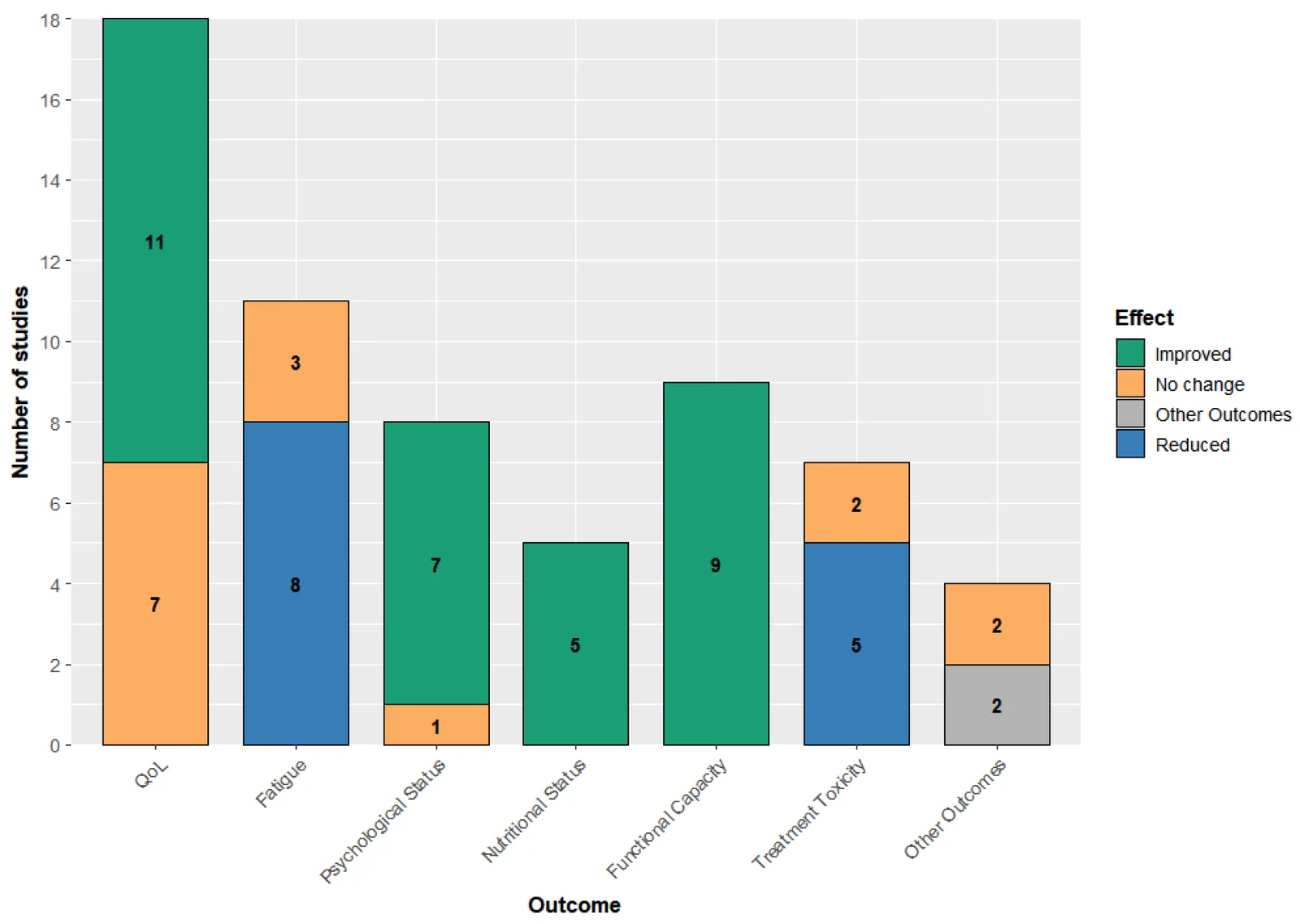
Reported outcomes and direction of effects of lifestyle interventions across the included studies.

The distribution of the available evidence according to the reported outcomes is presented in Figure 3. QoL was the most frequently assessed outcome (*n* = 18), followed by fatigue (*n* = 11), functional capacity (*n* = 9), psychological status (*n* = 8), and nutritional status (*n* = 5). Seven studies evaluated the effect of lifestyle interventions on treatment related toxicity, while four studies assessed other outcomes including sleep, pain severity, pain interference, anxiety, and coping. Among the two most frequently assessed outcomes, improvements in QoL were reported in 11 studies and reduction in fatigue in eight studies following lifestyle interventions. No significant improvement in QoL was reported in seven studies, while three found no significant reduction in fatigue. All studies assessing functional capacity or nutritional status reported improvements following lifestyle interventions. Improvements in psychological status were reported in seven studies whereas one found no significant change. Regarding treatment related toxicity, five studies reported a reduction following lifestyle intervention, where two found no significant change.

Outcome evaluations primarily relied on validated self-report tools, predominantly the EORTC questionnaire family (*n* = 12). Specifically, the QLQ-C30 was the most common instrument (*n* = 10), supplemented by additional EORTC modules (*n* = 3), as well as the SF-36 and FACT/FACIT scales (*n* = 2 each). Fatigue was measured using specialized scales (*n* = 6) such as FACIT-F, the Piper Fatigue Scale, and the Brief Fatigue Inventory (*n* = 2). Psychological status was assessed via the HADS (*n* = 3) and the Distress Thermometer (*n* = 2). Treatment toxicity was documented using CTCAE and RTOG criteria, while nutritional assessment relied mainly on the PG-SGA (*n* = 4) alongside biochemical markers. The above measures were complemented by physical capacity tests (*n* = 5), most notably the 6-Minute Walk Test (6MWT) (*n* = 3).

### 3.3. Synthesis of the Results

**Figure 4:**
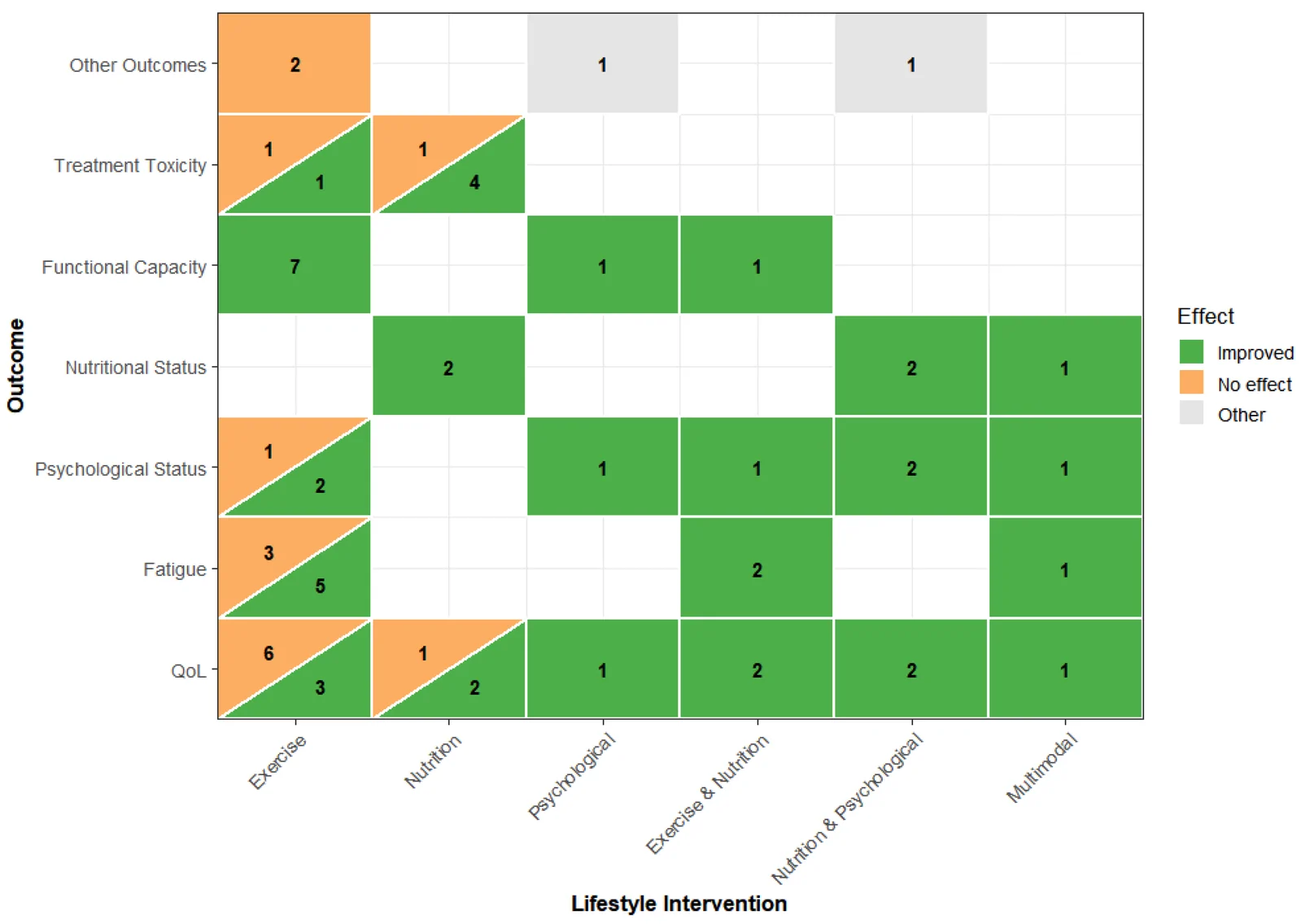
Evidence gap map summarizing the direction of clinical effects of lifestyle interventions across outcome domains. Green tiles indicate studies reporting improved outcomes, orange tiles indicate no significant effect, and grey tiles represent other outcomes that could not be classified as either improvement or no effect. The counts within each tile represent the number of study-outcome pairs (i.e., a single study may contribute to multiple outcome domains). Diagonally divided tiles indicate mixed findings within a given intervention–outcome pairing.

The effects of lifestyle interventions across the assessed outcomes are summarized in Figure 4. The distribution of studies and reported effects varied across interventions and outcome domains. Overall, the evidence map highlights heterogeneity in both the volume of evidence and the direction of reported effects.

#### 3.3.1. Exercise Interventions

Exercise interventions were heterogeneous and included structured aerobic and resistance-training programs (*n* = 2) [11,16], supervised moderate-intensity physical exercise programs (*n* = 2) [24,26], and dance-movement therapy (*n* = 2) [17,19]. One study compared two exercise interventions—high-intensity interval training (HIIT) and resistance training—with usual care [28]. Other interventions included a physical rehabilitation program incorporating quadriceps electrical stimulation and cycle ergometer training [14], a home-based graduated walking program [22], a general physical-exercise program [33], yoga [18], and qigong/tai chi [25].

**Figure 5:**
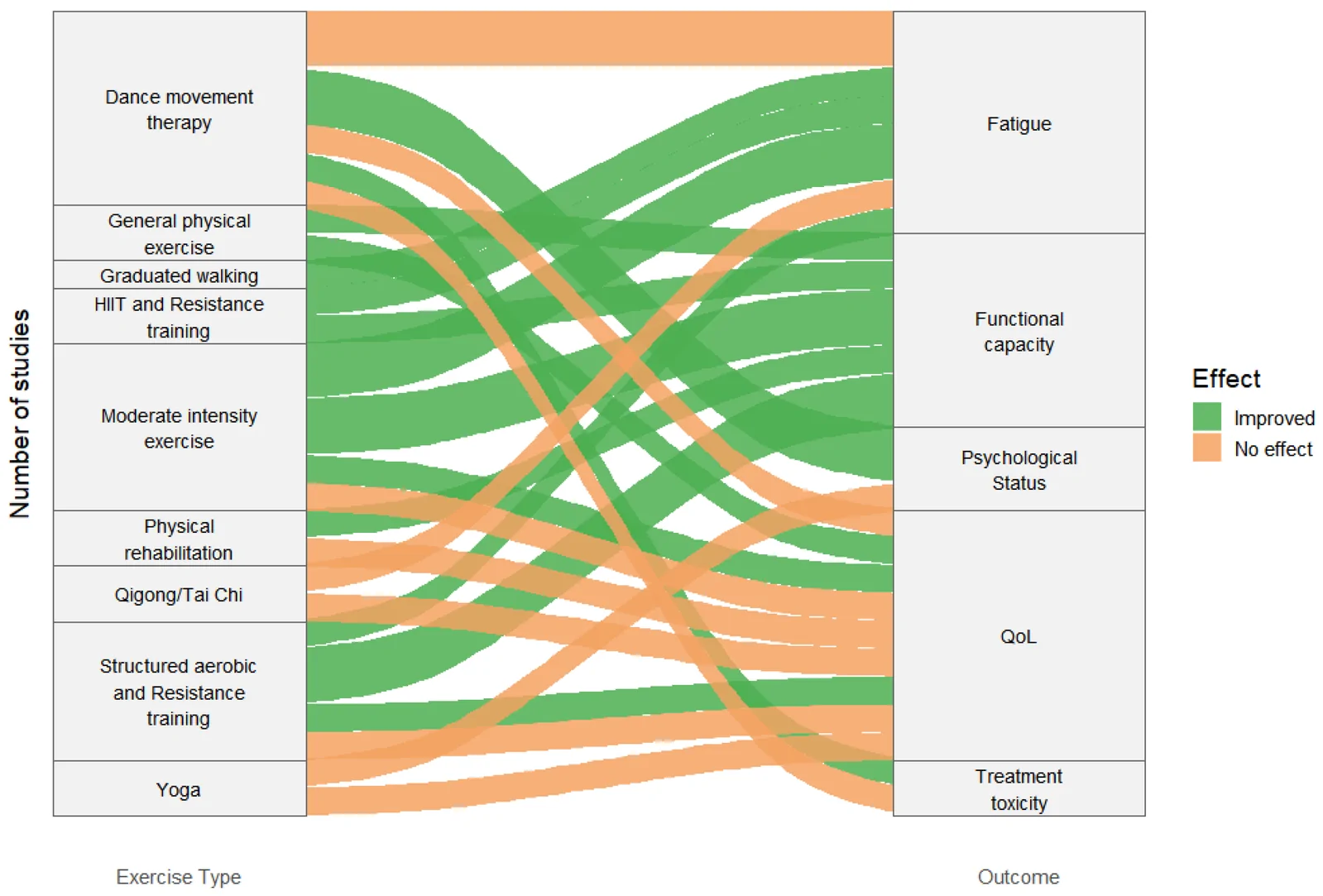
Sankey diagram of exercise interventions and associated outcomes. Flow width reflects number of studies; color indicates effect (green = improved, orange = no effect).

The effects of exercise interventions across outcome domains are illustrated in Figure 5. Structured aerobic and resistance training has been primarily associated with improvements in functional capacity and fatigue, while mixed findings were reported for QoL. Moderate-intensity exercise interventions were consistently associated with improvements in functional capacity and fatigue, with additional improvements in QoL reported in some studies. Physical rehabilitation interventions, incorporating quadriceps electrical stimulation and cycle ergometer training, were associated with improvements in functional capacity and no effect QoL. Dance movement therapy showed improvements on psychological status, no effect on fatigue and QoL, and mixed findings on treatment related toxicity. Yoga demonstrated no significant effect on psychological status or QoL, while qigong/tai chi showed no effect on fatigue and QoL. HIIT combined with resistance training was associated with improvements in fatigue and functional capacity. A home-based graduated walking program was associated with improvements in fatigue, whereas a general physical exercise program was associated with improved functional capacity and QoL.

#### 3.3.2. Nutrition Interventions

Nutrition interventions included evaluated tailored dietary regimens, specialized feeding protocols and modifications in fiber intake [13,27,29,30,31]. Nutrition intervention demonstrated clinically relevant benefits. Early nutritional intervention initiated at the start of RT in HNC patients reduced the incidence of high-grade oral mucositis and improved weight maintenance [13]. Similarly, a sequential nutrition intervention guided by nutritional risk screening effectively improves nutritional status and QoL while reducing complications in pancreatic cancer patients undergoing RT [31]. Dietary modification strategies also showed beneficial effects. A modified diet reduced the risk of constipation in CC patients [30], while a high-fiber diet was associated with reduced GI toxicity and improved QoL in mixed GYN and lower-GI cancer patients receiving RT in the pelvic area [29]. However, evidence was not entirely consistent, as fiber modification in PCa showed only marginal short-term benefits and was associated with increased abdominal bloating [27].

#### 3.3.3. Psychological Interventions

Psychological only interventions included nurse-led distress screening [21]. Nurse-led distress screening in BC patients was associated with improvements in QoL, functional capacity, and psychological status particularly in the subgroup receiving multimodality treatment (surgery and CRT) [21].

#### 3.3.4. Combined and Multimodal Interventions

Adapted Physical Activity and Diet (APAD) programs combining structured exercise and dietary modification were associated with reductions in fatigue, improvements in QoL and reductions in anxiety and depression [20,23]. Nutritional and psychological interventions have demonstrated benefits in patients undergoing RT [12,15]. Specifically, a combined nutritional and psychological intervention incorporating motivational interviewing and cognitive-behavioral therapy (Eating As Treatment) was associated with fewer RT interruptions, reduced weight loss, improved QoL and improved psychological status in patients with HNC [12]. Similarly, the supportive Holistic Interventions by Nurses and Experts via Multidisciplinary Team (SHINE-MDT), was also associated with improvements in QoL, nutritional status, and psychological status in a separate HNC population [15]. A multimodal nurse-led rehabilitation program combining exercise, nutrition, and psychological supportive care components was associated with improvements in QoL, psychological and nutritional status, and reductions in fatigue in patients with esophageal cancer [32].

## 4. Discussion

This scoping review mapped the available randomized evidence on lifestyle interventions delivered during RT and identified 23 RCTs evaluating exercise, nutritional, psychological, combined exercise–nutritional or nutritional–psychological, and multimodal interventions. A range of clinical and patient-reported outcomes were assessed, including QoL, fatigue, functional capacity, psychological status, nutritional status, and treatment related toxicities. The review revealed an uneven distribution of studies across intervention types and outcome assessments, and identified important gaps in the evidence on lifestyle interventions delivered during RT.

Exercise interventions represented the largest body of evidence reflecting the growing recognition of physical activity as a key component of supportive cancer care [34]. Interventions encompassed a broad range of modalities, including structured aerobic and resistance training, moderate-intensity exercise, physical rehabilitation, home-based walking programs, yoga, qigong/tai chi, dance movement therapy, and high-intensity interval training (Figure 5). Across these interventions, the most consistent benefits were observed for functional capacity, while evidence supporting improvements in QoL, treatment toxicity, and psychological related outcomes were less consistent. Structured aerobic and resistance training, moderate-intensity exercise, home-based walking, and high-intensity interval training programs were all associated with improvements in fatigue (Figure 5). Despite these encouraging findings, substantial variability in intervention protocols, duration, intensity, and delivery methods limit definitive conclusions regarding the optimal exercise prescription during RT and underscore the need for standardized protocols.

Nutritional, psychological, combined, and multimodal approaches remain comparatively underexplored. Although nutritional and combined interventions showed favorable findings on QoL, fatigue, nutritional status, and treatment toxicities in selected studies, the limited number of trials does not allow firm conclusions regarding optimal nutritional strategies or the comparative effectiveness of combined versus single-component interventions. Additionally, multimodal programs were associated with improvements in patient-reported outcomes, as well as reduced treatment interruptions and enhanced treatment tolerance, suggesting that comprehensive supportive care may offer additional benefits. This imbalance limits our understanding of the multidimensional nature of supportive oncology care and suggests that current evidence may not fully capture the potential benefits of comprehensive lifestyle modification.

The included RCTs were concentrated in BC, HNC, and PCa, whereas cervical, pancreatic, and esophageal cancers were represented by few studies. No RCTs involving patients with lung cancer or those with locally advanced or metastatic disease were identified. These populations frequently experience substantial cancer-related symptom burden, including fatigue, cachexia, and pain from bone metastases, while often requiring more intensive local and systemic treatments. Lifestyle interventions in these settings may be more challenging to implement and may carry additional safety considerations due to comorbidities, poor performance status, and metastases.

More than half of the available studies included patients receiving combined CRT. In addition, most studies did not report specific RT details, such as the treatment regimen, dose, fractionation schedule, or delivery technique, nor did they perform stratified analyses to examine whether intervention effects differed between conventional and hypofractionated RT, which have distinct toxicity profiles. Consequently, the independent contribution of supportive lifestyle interventions in patients undergoing RT alone remains unclear, and the potential influence of treatment-related factors on intervention outcomes cannot be adequately evaluated.

A gap that is also consistently highlighted in the literature is the lack of cost-effectiveness analyses. Healthcare resources remain limited, and the development of structured systems to support and coordinate these interventions requires substantial investment and organizational capacity. Given that lifestyle interventions do not consistently demonstrate clear or definitive improvements across all relevant outcomes, such analyses are essential to guide the efficient allocation of resources and to support the translation of evidence into clinical practice and health policy decision-making. Patient-centered supportive cancer care also depends on access to reliable, evidence-based patient information. High-quality web-based oncology resources can complement lifestyle interventions by supporting patient education and informed decision-making [35].

Several strengths and limitations of this review should be acknowledged. While scoping reviews typically include pilot and feasibility studies to comprehensively map the available evidence, this review was restricted to RCTs to provide a synthesis based on a consistent study design of the clinical effects of lifestyle interventions during RT. Consequently, early-stage evidence on intervention feasibility and implementation may not have been captured. Furthermore, much of the existing literature evaluated lifestyle interventions across multiple phases of the cancer treatment or survivorship without distinguishing outcomes specific to the RT treatment period. To address this issue, the context of the present review was restricted to interventions delivered during RT. For studies reporting outcomes before, during, and after RT, only data collected during the RT treatment period were extracted and synthesized. The included studies varied substantially in sample size, cancer populations, treatment protocols, and intervention components modality, intensity, frequency, duration, and delivery. Considerable heterogeneity was also observed in outcome assessment, with different validated instruments used to evaluate QoL, fatigue, psychological status, nutritional status, functional capacity, and treatment toxicities. This variability may have influenced interpretation of the evidence map and the identification of patterns across intervention types and outcomes. Consistent with scoping review methodology, no formal risk-of-bias assessment or quantitative synthesis was performed, as the objective was to map the available evidence and identify research gaps rather than assess intervention effectiveness. The publication-based evidence followed in this review may result in some overrepresentation of the available evidence when multiple publications arise from the same RCT (e.g., APAD trial [20,23]), irrespective of whether they involve the same or different cohorts. Despite efforts to develop a comprehensive search strategy, some eligible studies may have been missed if relevant outcomes were not captured by the specified search terms. The search was limited to PubMed and Web of Science; therefore, relevant studies indexed exclusively in other databases, such as Embase, CINAHL, PsycINFO, or Cochrane CENTRAL, may not have been identified. Finally, the search was restricted to English-language publications, which may have resulted in the omission of relevant studies published in other languages.

The findings of this review suggest a potential benefit rather than a definitive effectiveness of lifestyle interventions in supportive care during RT. Individualized, patient-centered approaches that consider treatment-related symptoms, functional status, nutritional needs, and psychosocial well-being may be most appropriate in current clinical practice.

## 5. Conclusions

This scoping review mapped the randomized evidence on lifestyle interventions delivered during RT and their effects on patient-reported and treatment-related outcomes. Exercise interventions comprised the largest body of evidence and were most consistently associated with improvements in fatigue and functional capacity, with less consistent effects on QoL. Nutritional, psychological, combined, and multimodal interventions were less frequently evaluated but showed favorable findings for selected outcomes. Important evidence gaps were identified, including limited evidence for non-exercise and multicomponent interventions, underrepresentation of several cancer populations, and heterogeneity in intervention characteristics and outcome measures. These gaps highlight the need for future RT-specific studies investigating a broader range of lifestyle interventions, including underrepresented cancer populations, and incorporating economic evaluations to better characterize their effects on treatment toxicity and patient-centered outcomes.

## Figures and Tables

**Table 1:** Characteristics and main outcomes of the included studies.

Author	Year	Cancer Type	Sample Size	Intervention	Duration (Weeks) ^1^	Tmt Type	Outcomes
Samuel et al. [11]	2019	HNC	148	E	11	CRT	Reduced fatigue; Improved QoL; Improved functional capacity
Britton et al. [12]	2019	HNC	307	N&P	6	CRT	Improved QoL; Improved nutritional status (smaller percentage of weight loss), fewer treatment interruptions, fewer unplanned hospital admissions and improved psychological status
Wei et al. [13]	2024	HNC	100	N	6–7	CRT	Reduced oral mucositis; Improved nutritional status and QoL
Vinches et al. [14]	2025	HNC	70	E	12	CRT	Improved functional capacity; No QoL change
Pei et al. [15]	2025	HNC	233	N&P	6	CRT	Improved QoL and nutritional status; Reduced psychological distress
D’Silva et al. [16]	2025	HNC	80	E	7	CRT	Improved functional capacity; No QoL change
Ho et al. [17]	2016	BC	139	E	3–6	RT	No change in fatigue or QoL; Reduced pain severity and pain interference; Improved psychological status
Ratcliff et al. [18]	2016	BC	163	E	6	RT	No change in QoL and Psychological Status
Ho et al. [19]	2018	BC	121	E	3	RT	No significant changes in fatigue or sleep; Reduced stress
Carayol et al. [20]	2019	BC	143	E&N	26	CRT	Reduced fatigue; Improved functional capacity, psychological status and QoL;
Ploos van Amstel et al. [21]	2020	BC	194	P	6	CRT	Improved QoL, Functional capacity and Psychological Status; Improved coping, and lower anxiety only for the multimodality treatment subgroup
VanderWalde et al. [22]	2021	BC	54	E	4–6	RT	Reduced fatigue
Touraine et al. [23]	2025	BC	143	E&N	26	CRT	Improved QoL; Reduced fatigue
Hojan et al. [24]	2016	PCa	54	E	6–7	RT	Reduced fatigue; Improved functional capacity and QoL
McQuade et al. [25]	2016	PCa	76	E	6–8	RT	No significant changes in fatigue, sleep, or QoL
Hojan et al. [26]	2017	PCa	72	E	50	RT	Improved fatigue and functional capacity; No QoL change
Forslund et al. [27]	2020	PCa	180	N	7	RT	No change in RT toxicity and QoL
Piraux et al. [28]	2021	PCa	72	E	5–8	RT	Reduced fatigue; Increased functional capacity
Wedlake et al. [29]	2017	Mixed -GYN; Lower-GI	166	N	5–7	CRT	Reduced gastrointestinal toxicity
Flores-Cisneros et al. [30]	2025	CC	134	N	5	CRT	Reduced gastrointestinal toxicity
Wang et al. [31]	2025	Pancreatic	100	N	4	CRT	Reduced RT toxicity; Improved QoL and Nutritional status
Chen et al. [32]	2025	Esophageal	70	Multimodal	4	RT	Reduced fatigue; Improved QoL and nutritional status; Improved Psychological status
Malik et al. [33]	2018	HNC;BC; CC; PCa	100	E	12	RT	Improved QoL; Enhanced functional capacity

^1^ Duration refers to the duration of the intervention or, if not explicitly stated in the referenced study, to the corresponding radiotherapy duration. Abbreviations: Tmt: Treatment; CRT: Chemoradiotherapy; RT: Radiotherapy; HNC: Head and Neck Cancer; BC: Breast Cancer; CC: Cervical Cancer; PCa: Prostate Cancer; GYN: Gynecological Cancer; Lower-GI: Gastrointestinal Cancer of the lower abdomen; E: Exercise; N: Nutrition; P: Psychological; QoL: Quality of Life.

## Data Availability

No new data were created or analyzed in this study. Data sharing is not applicable to this article.

## Supplementary Materials

The following supporting information can be downloaded at: https://www.mdpi.com/article/10.3390/medsci14040501/s1, Table S1: PRISMA-ScR Checklist.

## Abbreviations

The following abbreviations are used in this manuscript:

QoL	Quality of Life
BC	Breast Cancer
HNC	Head and Neck Cancer
PCa	Prostate Cancer
CC	Cervical Cancer
GI	Gastrointestinal
RT	Radiotherapy
CHT	Chemotherapy
CRT	Chemoradiotherapy
PCC	Population-Concept-Context
RCT	Randomized Clinical Trial

## References

[1] Bray F., Laversanne M., Sung H., Ferlay J., Siegel R.L., Soerjomataram I., Jemal A. (2024). Global Cancer Statistics 2022: GLOBOCAN Estimates of Incidence and Mortality Worldwide for 36 Cancers in 185 Countries. CA Cancer J. Clin..

[2] Zhu H., Chua M.L.K., Chitapanarux I., Kaidar-Person O., Mwaba C., Alghamdi M., Rodríguez Mignola A., Amrogowicz N., Yazici G., Bourhaleb Z. (2024). Global Radiotherapy Demands and Corresponding Radiotherapy-Professional Workforce Requirements in 2022 and Predicted to 2050: A Population-Based Study. Lancet Glob. Health.

[3] Feighan L., MacDonald-Wicks L., Callister R., Surjan Y. (2024). The Effectiveness of Exercise and/or Nutritional Interventions to Improve the Quality of Life of Women with Breast Cancer Receiving Radiation Therapy: A Scoping Review. Support. Care Cancer.

[4] Briggs L.G., Parke S.C., Gill V.S., Van Ligten M.J., Beck K.L., Sinha D., Alkhatib K.Y., Choudry M.M., Bain P.A., Quillen J. (2026). Exercise, Nutrition, and Psychological Support for Kidney Cancer: A Scoping Review. BJU Int..

[5] Chalmers O., Waddell A., Choudhury A., Sale C., Harwood A.E. (2025). Elucidating the Mechanisms of Lifestyle Interventions in Mitigating Radiotherapy Adverse Effects: A Scoping Review. BMJ Oncol..

[6] Zuo S., Cheng H., Wang Z., Liu T., Chen S., Tian L., Lin L. (2023). Nonpharmacological Interventions for Cancer-Related Fatigue: A Literature Review. Asia-Pac. J. Oncol. Nurs..

[7] Kumar B., Htaa M.T., Kerin-Ayres K., Smith A.L., Lacey J., Browne S.B., Grant S. (2024). Living Well with Advanced Cancer: A Scoping Review of Non-Pharmacological Supportive Care Interventions. J. Cancer Surviv..

[8] Peters M.D., Godfrey C., McInerney P., Munn Z., Tricco A.C., Khalil H. (2024). Scoping Reviews. JBI Manual for Evidence Synthesis.

[9] Peters M.D.J., Marnie C., Tricco A.C., Pollock D., Munn Z., Alexander L., McInerney P., Godfrey C.M., Khalil H. (2020). Updated Methodological Guidance for the Conduct of Scoping Reviews. JBI Evid. Synth..

[10] Tricco A.C., Lillie E., Zarin W., O’Brien K.K., Colquhoun H., Levac D., Moher D., Peters M.D.J., Horsley T., Weeks L. (2018). PRISMA Extension for Scoping Reviews (PRISMA-ScR): Checklist and Explanation. Ann. Intern. Med..

[11] Samuel S.R., Maiya A.G., Fernandes D.J., Guddattu V., Saxena P.P., Kurian J.R., Lin P., Mustian K.M. (2019). Effectiveness of Exercise-Based Rehabilitation on Functional Capacity and Quality of Life in Head and Neck Cancer Patients Receiving Chemo-Radiotherapy. Support. Care Cancer.

[12] Britton B., Baker A.L., Wolfenden L., Wratten C., Bauer J., Beck A.K., McCarter K., Harrowfield J., Isenring E., Tang C. (2019). Eating As Treatment (EAT): A Stepped-Wedge, Randomized Controlled Trial of a Health Behavior Change Intervention Provided by Dietitians to Improve Nutrition in Patients With Head and Neck Cancer Undergoing Radiation Therapy (TROG 12.03). Int. J. Radiat. Oncol. Biol. Phys..

[13] Wei J., Chen Y., Su J., Zhao Q., Wang H., Zheng Z., Wu J., Jiang X. (2024). Effects of Early Nutritional Intervention on Oral Mucositis and Basic Conditions in Patients Receiving Radiotherapy for Head and Neck Cancer: Randomized Controlled Trial (ChiCTR2000031418). Clin. Nutr..

[14] Vinches M., Janiszewski C., Thezenas S., Delample D., Boisselier P., Jacquot S., Senesse P., Faravel K. (2026). Evaluation of Physical Rehabilitation in Patients Receiving Curative Treatment for Head and Neck Cancer: The NUTRIMOUV Randomized Phase II Trial. Support. Care Cancer.

[15] Pei Y., Wang J., Li J., Chen Y., He J., Wei Z., Liu Z., Su Y., Dai T., Yin L. (2025). Multidisciplinary Team Support for Patients with Head and Neck Cancer Receiving Radiotherapy: A Randomized Clinical Trial. JAMA Netw. Open.

[16] D’Silva C., Shetty V., Fernandes D., Jean-Pierre B., Kumari S., Srivastava S., Samuel S.R. (2025). Effect of Structured Exercise-Based Rehabilitation on Sarcopenia and Quality of Life among Head and Neck Cancer Patients Undergoing Chemo-Radiotherapy: A Randomized Controlled Trial. Asian Pac. J. Cancer Prev..

[17] Ho R.T.H., Fong T.C.T., Cheung I.K.M., Yip P.S.F., Luk M.Y. (2016). Effects of a Short-Term Dance Movement Therapy Program on Symptoms and Stress in Patients with Breast Cancer Undergoing Radiotherapy: A Randomized, Controlled, Single-Blind Trial. J. Pain Symptom Manag..

[18] Ratcliff C.G., Milbury K., Chandwani K.D., Chaoul A., Perkins G., Nagarathna R., Haddad R., Nagendra H.R., Raghuram N.V., Spelman A. (2016). Examining Mediators and Moderators of Yoga for Women with Breast Cancer Undergoing Radiotherapy. Integr. Cancer Ther..

[19] Ho R.T.H., Fong T.C.T., Yip P.S.F. (2018). Perceived Stress Moderates the Effects of a Randomized Trial of Dance Movement Therapy on Diurnal Cortisol Slopes in Breast Cancer Patients. Psychoneuroendocrinology.

[20] Carayol M., Ninot G., Senesse P., Bleuse J.-P., Gourgou S., Sancho-Garnier H., Sari C., Romieu I., Romieu G., Jacot W. (2019). Short- and Long-Term Impact of Adapted Physical Activity and Diet Counseling during Adjuvant Breast Cancer Therapy: The “APAD1” Randomized Controlled Trial. BMC Cancer.

[21] Ploos van Amstel F.K., Peters M.E.W.J., Donders R., Schlooz-Vries M.S., Polman L.J.M., van der Graaf W.T.A., Prins J.B., Ottevanger P.B. (2020). Does a Regular Nurse-Led Distress Screening and Discussion Improve Quality of Life of Breast Cancer Patients Treated with Curative Intent? A Randomized Controlled Trial. Psychooncology.

[22] VanderWalde N.A., Martin M.Y., Kocak M., Morningstar C., Deal A.M., Nyrop K.A., Farmer M., Ballo M., VanderWalde A., Muss H. (2021). Randomized Phase II Study of a Home-Based Walking Intervention for Radiation-Related Fatigue among Older Patients with Breast Cancer. J. Geriatr. Oncol..

[23] Touraine C., Jacot W., Gourgou S., Carayol M., Senesse P., Ninot G., Mollevi C. (2025). Impact of Adapted Physical Activity and Diet Counselling on Health-Related Quality of Life in Women Undergoing Adjuvant Breast Cancer Therapy. Sci. Rep..

[24] Hojan K., Kowska-Borowczyk E.K., Leporowska E., Gorecki M., Ozga-Majchrzak O., Milecki T., Milecki P. (2016). Physical Exercise for Functional Capacity, Blood Immune Function, Fatigue, and Quality of Life in High-Risk Prostate Cancer Patients during Radiotherapy: A Prospective, Randomized Clinical Study. Eur. J. Phys. Rehabil. Med..

[25] McQuade J.L., Prinsloo S., Chang D.Z., Spelman A., Wei Q., Basen-Engquist K., Harrison C., Zhang Z., Kuban D., Lee A. (2017). Qigong/Tai Chi for Sleep and Fatigue in Prostate Cancer Patients Undergoing Radiotherapy: A Randomized Controlled Trial. Psychooncology.

[26] Hojan K., Kwiatkowska-Borowczyk E., Leporowska E., Milecki P. (2017). Inflammation, Cardiometabolic Markers, and Functional Changes in Men with Prostate Cancer: A Randomized Controlled Trial of a 12-Month Exercise Program. Pol. Arch. Intern. Med..

[27] Forslund M., Ottenblad A., Ginman C., Johansson S., Nygren P., Johansson B. (2020). Effects of a Nutrition Intervention on Acute and Late Bowel Symptoms and Health-Related Quality of Life up to 24 Months Post Radiotherapy in Patients with Prostate Cancer: A Multicentre Randomised Controlled Trial. Support. Care Cancer.

[28] Piraux E., Caty G., Renard L., Vancraeynest D., Tombal B., Geets X., Reychler G. (2021). Effects of High-Intensity Interval Training Compared with Resistance Training in Prostate Cancer Patients Undergoing Radiotherapy: A Randomized Controlled Trial. Prostate Cancer Prostatic Dis..

[29] Wedlake L., Shaw C., McNair H., Lalji A., Mohammed K., Klopper T., Allan L., Tait D., Hawkins M., Somaiah N. (2017). Randomized Controlled Trial of Dietary Fiber for the Prevention of Radiation-Induced Gastrointestinal Toxicity during Pelvic Radiotherapy. Am. J. Clin. Nutr..

[30] Flores-Cisneros L., Cetina-Pérez L., Luvián-Morales J., Galicia-Carmona T., Jiménez-Morales R., Sánchez-López M., Castillo-Martínez L. (2025). Fiber, Lactose and Fat-Modified Diet for the Prevention of Gastrointestinal Chemo-Radiotherapy-Induced Toxicity in Patients with Cervical Cancer: Randomized Clinical Trial. Nutrition.

[31] Wang F., Zhang L., Li N., Zhao D., Su X., Liu J., Shen X., Zhang S., Fang H., Wang C. (2025). Sequential Nutrition Intervention Guided by Nutritional Risk Screening Improves Outcomes Following CyberKnife Radiotherapy for Pancreatic Cancer: A Randomized Controlled Trial. Pancreatology.

[32] Chen X., Zhong L., Wang J., Dai T., Li M., Luo J., Zhu L., Liu X., Tao L., Li L. (2025). Multimodal Rehabilitation to Improve Quality of Life and Fatigue after Radiotherapy in Esophageal Cancer Patients: A Randomized Trial. Oncologist.

[33] Malik Y., Sen J., Mishra A., Bhandari V. (2023). Effects of Physical Exercise on Rehabilitation of Cancer Patients Undergoing Radiotherapy. J. Cancer Res. Ther..

[34] Avancini A., Borsati A., Toniolo L., Ciurnelli C., Belluomini L., Budolfsen T., Lillelund C., Milella M., Quist M., Pilotto S. (2025). Physical Activity Guidelines in Oncology: A Systematic Review of the Current Recommendations. Crit. Rev. Oncol. Hematol..

[35] Fionda B., Piras A., D’Aviero A., Venuti V., Casà C., Preziosi F., Catucci F., Boldrini L., Daidone A., Tagliaferri L. (2021). The “PC-WIRED” Study: Patient Centred Evolution of Websites of Italian Radiotherapy Departments. Patient Educ. Couns..

